# Therapeutic Options Targeting Oxidative Stress, Mitochondrial Dysfunction and Inflammation to Hinder the Progression of Vascular Complications of Diabetes

**DOI:** 10.3389/fphys.2018.01857

**Published:** 2019-01-17

**Authors:** João S. Teodoro, Sara Nunes, Anabela P. Rolo, Flávio Reis, Carlos M. Palmeira

**Affiliations:** ^1^Center for Neurosciences and Cell Biology, Department of Life Sciences, University of Coimbra, Coimbra, Portugal; ^2^Laboratory of Pharmacology and Experimental Therapeutics, Faculty of Medicine, Coimbra Institute for Clinical and Biomedical Research, University of Coimbra, Coimbra, Portugal

**Keywords:** type 2 diabetes mellitus, oxidative stress, mitochondrial dysfunction, inflammation, diabetic vascular complications, therapeutics

## Abstract

Type 2 diabetes mellitus is a leading cause of morbidity and mortality worldwide, given its serious associated complications. Despite constant efforts and intensive research, an effective, ubiquitous treatment still eludes the scientific community. As such, the identification of novel avenues of research is key to the potential discovery of this evasive “silver bullet.” We focus on this review on the matter of diabetic injury to endothelial tissue and some of the pivotal underlying mechanisms, including hyperglycemia and hyperlipidemia evoked oxidative stress and inflammation. In this sense, we revisited the most promising therapeutic interventions (both non-pharmacological and antidiabetic drugs) targeting oxidative stress and inflammation to hinder progression of vascular complications of diabetes. This review article gives particular attention to the relevance of mitochondrial function, an often ignored and understudied organelle in the vascular endothelium. We highlight the importance of mitochondrial function and number homeostasis in diabetic conditions and discuss the work conducted to address the aforementioned issue by the use of various therapeutic strategies. We explore here the functional, biochemical and bioenergetic alterations provoked by hyperglycemia in the endothelium, from elevated oxidative stress to inflammation and cell death, as well as loss of tissue function. Furthermore, we synthetize the literature regarding the current and promising approaches into dealing with these alterations. We discuss how known agents and therapeutic behaviors (as, for example, metformin, dietary restriction or antioxidants) can restore normality to mitochondrial and endothelial function, preserving the tissue’s function and averting the aforementioned complications.

## Introduction

Type 2 diabetes mellitus (T2DM) is one of the 21st century’s global public health problems, with estimates of the affected population reaching 425 million people (IDF, 2017). The global prevalence of diabetes is rapidly growing in such a way that, according to the International Diabetes Federation (IDF) estimates, by 2045 a total of 629 million people will have diabetes (IDF, 2017). The increase in diabetes prevalence is driven in large part by increasing rates of obesity and aging of the global population as well as changes in lifestyle related to unhealthy eating habits and sedentarism, with significant costs to healthcare systems. In 2017, diabetes caused more than 477 thousand deaths among adults in Europe alone; 32.9% of these deaths were of people under the age of 60 (IDF, 2017). In Portugal, according to data from the Annual Report of the Portuguese National Diabetes Observatory, the estimated prevalence of diabetes in 2015 was 13.3% (out of a population of 7.7 million individuals aged 20–79 years), meaning that more than 1 million Portuguese citizens in this age group have diabetes, of which 5.8% remain undiagnosed (Sociedade Portuguesa de Diabetologia, 2016). Considering the constantly increasing socio-economic and public health impact of diabetes and its complications, it is therefore critical to identify the underlying causes of diabetes to discover further measures of effective prevention and treatment (Tol et al., 2013).

Type 2 diabetes mellitus is a complex metabolic disorder associated with hyperglycemia, caused by defects in insulin secretion and/or action (Bergman, 2013; Alam et al., 2014). Over time, hyperglycemia induces toxic effects in virtually all of the organs of the body, of which the vascular system is particularly affected, resulting in multiple complications either at the microvascular level (retinopathy, nephropathy and neuropathy) or at the macrovascular level (stroke, coronary heart disease, acute myocardial infarction and peripheral vascular disease) (Calcutt et al., 2009; Gray and Jandeleit-Dahm, 2014; Heinonen et al., 2015). Approximately 40% of people with diabetes have late complications resulting from their disease progressing silently before the diagnosis is performed or even completed (IDF, 2015).

The pathophysiology of the link between T2DM and vascular complications is complex and multi-factorial. In fact, the precise mechanism by which T2DM leads to the development of these complications is complex and not yet fully elucidated, but seems to be strongly related with the toxic effects derived from hyperglycemia as well as by hyperlipidemia originated from obesity – gluco and lipo toxicity, respectively. Hyperglycemia induces oxidative stress, namely via mitochondrial dysfunction and enhanced reactive oxygen species (ROS) generation, while hyperlipidemia contributes to the release of pro-inflammatory cytokines by the adipocyte tissue. The consequent oxidative stress and low-grade-inflammation have been considered major contributors for the progression of T2DM and its complications (Santilli et al., 2015). In addition to the hyperglycemia and hyperlipidemia-induced toxicity, insulin resistance and hypertension promote damage at the level of blood vessel walls, which are manifested through the development of endothelial dysfunction (van den Oever et al., 2010), a condition that precedes the early development of micro and macrovascular diseases and complications (Cade, 2008).

Mitochondria play a key role in metabolic processes in all cells within an organism. Along with the famous ATP-generating oxidative phosphorylation, these organelles are critical for calcium chelation, biosynthetic pathways, ROS generation and cell death amongst many others. Unsurprisingly, interference and hampering of mitochondrial function is a hallmark of countless pathologic conditions and a central event of the progression of many diseases (Duchen, 2004). Depending on the endothelium location and functions, mitochondrial content of endothelial cells (EC) varies dramatically, as so does their function. Furthermore, their intracellular distribution is also reflective of their role within the cell (Park et al., 2011). As such, it is evident that mitochondrial dysfunction is at the core of endothelial injury, typically by inflammation, oxidative stress, cell death and loss of tissue function (Tang et al., 2014).

Even though there is a clear association between hyperglycemia and diabetic complications, the benefits of strict glycemic control on micro and macrovascular complications have been questioned, and interventions able to protect the organs targeted by diabetes are mandatory. Therapeutic strategies targeting oxidative stress, mitochondrial dysfunction and inflammation might be crucial to hinder the progression of vascular complications of diabetes. This review summarizes the contribution of the relationship between oxidative stress, mitochondrial oxidative damage and inflammatory aspects associated with endothelial dysfunction to the development of diabetes-related vascular complications. We also discuss therapeutic approaches that could prevent diabetic complications by specially targeting oxidative stress, mitochondrial impairment and inflammation pathways.

## The Crosstalk Between Oxidative Stress and Inflammation in the Progression of T2DM Complications

**FIGURE 1:**
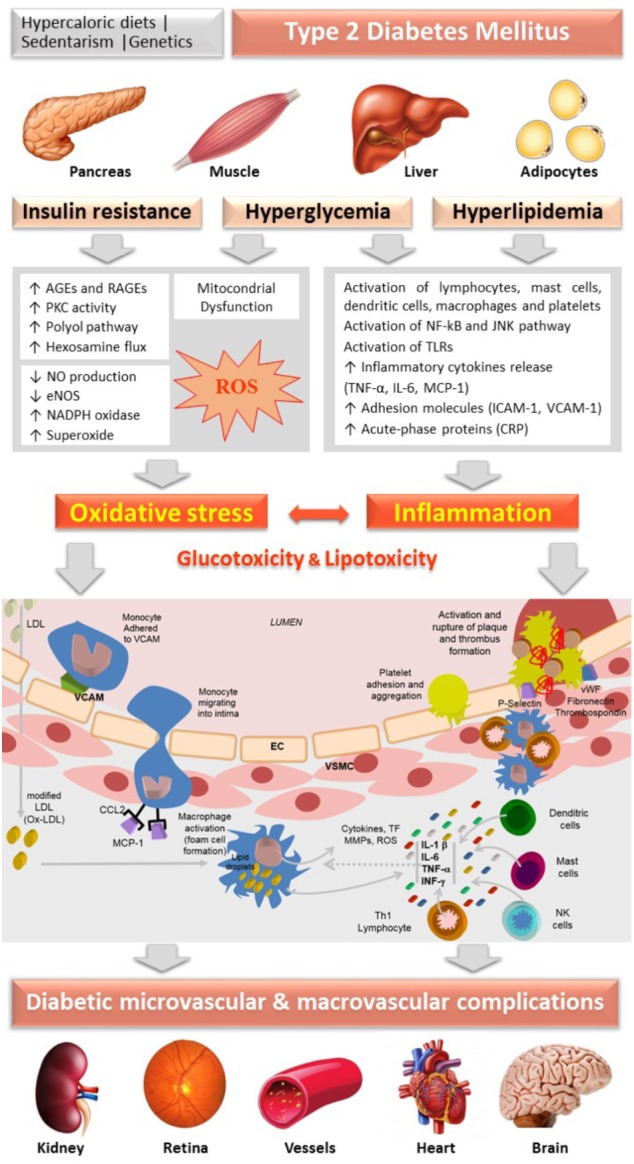
Schematic diagram representing the central role of oxidative stress and inflammation, guided by insulin resistance, hyperglycemia and hyperlipidemia (gluco and lipo toxicity), in the vascular changes underlying the progression of micro and macrovascular complications of diabetes. AGEs, advanced glycation end products; CRP, C-reactive-protein; EC, endothelial cells; eNOS, endothelial nitric oxide synthase; ICAM-1, intracellular adhesion molecule-1; IL, interleukin; IFN, interferon; JNK, c-jun NH2-terminal kinase; LDL, Low Density Lipoprotein; MCP-1 (CCL-2), Monocyte chemotactic protein-1; NF-κB, nuclear factor-κB; NO, nitric oxide; PKC, protein kinase C; RAGEs, receptor for AGEs; ROS, reactive oxygen species; TLR, Toll-Like Receptor; TNF-α, tumor necrosis factor-alfa; VSMC, vascular smooth muscle cells; VCAM-1, vascular cell adhesion molecule; vWF, von Willebrand factor.

Diabetic patients frequently have several risk factors for development of vascular complications, including age, insulin resistance, dyslipidemia, hyperglycemia and hypertension. T2DM is not solely a metabolic disease but also a vascular one, characterized by chronic hyperglycemia and alterations of cellular homeostasis leading to vascular complications. The link between insulin resistance/hyperglycemia and endothelial dysfunction plays an important role in the development and progression of atherosclerotic disease in T2DM (Onat et al., 2006; Hadi and Suwaidi, 2007). Growing evidence suggests that hyperglycemia-induced oxidative stress promotes endothelial dysfunction by increased production of ROS, which plays a major role in the pathogenesis and progression of diabetic vascular complications (Pitocco et al., 2013; Figure 1).

Hyperglycemia, associated with insulin resistance and excessive free fatty acids (FFAs), initiates diabetic vascular complications through many metabolic and structural derangements, including in endothelial and vascular smooth muscle cells (VSMCs), compromising the vascular physiology and function. There are several proposed mechanisms underlying this hyperglycemia-evoked vascular damage, including increased production of advanced glycation end products (AGEs) and expression of their receptors RAGE (Receptor for AGEs), activation of protein kinase C (PKC) isoforms, as well as increased activation of the polyol and hexosamine fluxes, which induce increased mitochondrial ROS production, non-enzymatic glycation of proteins and auto-oxidation of glucose. The activation of these pathways resulting from prolonged hyperglycemia induces glucotoxicity (Brownlee, 2001; Rolo and Palmeira, 2006).

The activation of the polyol pathway contributes to oxidative stress as it causes nicotinamide adenine dinucleotide phosphate (NADPH) depletion at the consequent decrease in intracellular reduced glutathione levels (Pitocco et al., 2013). In an environment of hyperglycemia, aldose reductase reduces glucose to sorbitol, which is subsequently oxidized to fructose. In the process of reducing the high intracellular glucose content, aldose reductase consumes NADPH, which is the cell’s main reducing agent and is also essential for the regeneration of the intracellular antioxidant glutathione (Brownlee, 2001, 2005).

Advanced glycation end products are generated by non-enzymatic glycosylation of proteins or lipids after prolonged exposure to glucose (Tamura et al., 2003). The formation of AGEs contributes to oxidative stress in several cell types through the interaction with their receptor, mainly by activation of NADPH oxidase and by the activation of the nuclear transcription factor kappa-B (NF-kB) pathway, leading to inflammatory and thrombogenic alterations that contribute to the pathogenesis of vascular diabetic complications (Brownlee, 2005; Giacco and Brownlee, 2010; Yamagishi et al., 2012). These glycated proteins accumulate on the vessels’ walls exposed to hyperglycemia, altering the structural integrity of the vascular wall and neutralizing nitric oxide (NO), substantially affecting endothelial function. The production of AGEs is also responsible for the decrease in endothelial NO synthase (eNOS) expression as well as macrophage-mediated inflammation in the vessels’ walls. In addition, they increase endothelin-1 (ET-1), an endothelium-derived potent vasoconstrictor, in EC (Santilli et al., 2015). Furthermore, circulating AGEs appear to react directly with lipoproteins, especially low-density lipoproteins (LDL), inducing structural alterations and damaging the mechanisms of LDL-receptor-mediated particle removal at tissue level (Bucala et al., 1994). Indeed, these products have been identified in atherosclerotic plaques, suggesting a possible role of AGEs in the pathophysiological of cardiovascular complications in diabetic patients (Figure 1).

The activation of PKC contributes to the production of superoxide anion in vascular EC (Ceriello et al., 2004; Paneni et al., 2013). The hyperglycemic environment causes chronic elevation of diacylglycerol (DAG) levels in EC, with membrane translocation of PKC isoforms. PKC phosphorylates NADPH oxidase, stimulating the production of superoxide, further aggravating oxidative stress (Paneni et al., 2013). This activation interferes with the biosynthesis of NO by decreasing eNOS activity through increasing the phosphorylation, also leading to an increment of ET-1 production, promoting increased vasoconstriction and platelet aggregation. In addition, it stimulates the synthesis of the extracellular matrix and promotes an inflammatory response through the activation of cytokines and adhesion molecules (Giacco and Brownlee, 2010; Paneni et al., 2013; Domingueti et al., 2016; Figure 1).

Another pathway that involves ROS generation is the production of NO, which plays a central role in the modulation of endothelial function, regulating vascular homeostasis and the innate immune system. In addition, NO generation by macrophages is elevated in various inflammatory conditions, including atherosclerosis (Bashan et al., 2009; Pitocco et al., 2013; Lundberg et al., 2015). The vasoprotective beneficial actions of NO include vasodilatation, increase in blood flow, hypotension, inhibition of platelet aggregation and adhesion, as well as reduction of smooth muscle proliferation (Toda et al., 2010). Endothelial dysfunction refers to an imbalance in the release of NO or other vasodilatory factors and vasoconstrictor substances, and is related to the pathology of diabetes and related complications (Ouviña et al., 2001). Under diabetic conditions, endothelial dysfunction leads to impaired NO availability, namely by eNOS uncoupling, leading to its deficiency and increase in vascular resistance, contributing to atherogenesis (Johnson, 2012). The reduction in vascular NO bioavailability is related to its inactivation by ROS, in such a way that has been used as a biomarker of oxidative stress (Pitocco et al., 2010).

In T2DM, the main sources of oxidative stress are the mitochondria (Rolo and Palmeira, 2006; Asmat et al., 2016) (details are discussed in the next section). Briefly, the increase in the generation of mitochondrial ROS has been implicated as a mediator between hyperglycemia and its pathological consequences in the vessels, kidneys, neurons and retina (Bashan et al., 2009). Glucose can directly stimulate the overproduction of ROS, which leads to the activation of several enzymatic cascades resulting in mitochondrial dysfunction, including activation of NADPH oxidase, decoupling of NO synthases and stimulation of xanthine oxidase (Giacco and Brownlee, 2010; Pitocco et al., 2013).

Insulin resistance is also associated with endothelial dysfunction, resulting in a reduction of biosynthesis and biological activity of NO (Tessari et al., 2010). Endothelial dysfunction caused by insulin resistance may also be provoked by the activation of the extracellular-signal-regulated kinase/mitogenic protein kinase (MAPK) pathways, which is responsible for insulin signaling. Insulin is also responsible for modulating the activity of eNOS through the phosphatidylinositol-3-kinase/serine-threonine kinase (PI3K/AKT) pathway (Sena et al., 2013). Thus, it has been demonstrated that subjects that present a reduction in eNOS expression are more susceptible to developing insulin resistance (Capellini et al., 2010).

In recent years, several reports have indicated that oxidative stress plays a crucial role in the development of inflammatory state. These pathological conditions reinforce each other, establishing a vicious cycle capable of extending and propagating the inflammatory response, contributing to the pathogenesis of T2DM and several other diseases (Lugrin et al., 2014). Moreover, mediators of inflammation have been associated with T2DM progression, and patients newly diagnosed with this condition have high levels of acute-phase proteins and proinflammatory cytokines, when compared to non-diabetic subjects (Yu et al., 2011; Nunes et al., 2012; Akash et al., 2013). Furthermore, chronic low-grade inflammation associated with states of obesity plays an important role in the development of chronic complications of diabetes (Nunes et al., 2012).

Gluco and lipo toxicity induce inflammation by several mechanisms, including activation of NF-kB, which leads to recruitment and activation of immune cells (Aminzadeh et al., 2013). ROS-induced expression of adhesion molecules, such as intracellular adhesion molecule-1 (ICAM-1) and vascular adhesion molecule-1 (VCAM-1), results in inflammatory cells’ recruitment (Kaneto et al., 2010). ICAM-1 is expressed in both the vascular endothelium and monocytes and, therefore, is considered a biomarker of both endothelial dysfunction and low-grade inflammation (Hubbard and Rothlein, 2000). Changes in NO, cytokines, acute-phase reactants and cellular adhesion molecules induced by the overproduction of ROS precede atherosclerosis (Figure 1).

Increased inflammation promotes increased migration of neutrophils and monocytes, causing increased production and release of cytokines and mediators of inflammation, such as interleukin-6 (IL-6) and tumor necrosis factor-alpha (TNF-α) as well as monocyte chemotactic protein 1 (MCP-1), among other pro-inflammatory proteins (Pedicino et al., 2013). C-reactive protein (CRP) is considered an acute phase inflammatory protein, synthesized by hepatocytes and predominantly regulated by IL-6 and TNF-α, thus being considered a sensitive and reliable marker of inflammatory state. Increased serum levels of CRP are also present in chronic inflammatory conditions such as atherosclerosis. Their levels are approximately tripled in the presence of risk of peripheral vascular diseases. CRP promotes the expression of adhesion molecules (ICAM-1 and VCAM-1), which facilitate the adhesion of monocytes and T cells to the arterial wall in the first steps of the atherogenic process. The high plasma concentrations of CRP increase the risk of cardiovascular events (peripheral vascular disease, myocardial infarction, stroke and death), even among adults who did not present previous chronic processes (Willerson and Ridker, 2004).

Increased levels of pro-inflammatory cytokines, including TNF-α, IL-6 and CRP, also contribute to the reduction of endothelial relaxation factor NO and to increased activity of ET-1, which causes vasoconstriction by targeting VSMCs. Thus, low-grade inflammation has been linked to increased vascular permeability, altered vasoregulatory responses and the adhesion of monocytes, neutrophils, and macrophages, resulting in cell damage. In addition, cytokine release stimulates the expression of plasminogen activator inhibitor type-1 (PAI-1), a pro-thrombotic protein associated with vascular homeostasis (van den Oever et al., 2010; van Hinsbergh, 2012). Elevated levels of PAI-1 may also impair fibrinolysis in patients with T2DM (Pandolfi et al., 2001).

The excess of FFAs influences the development of insulin resistance through the activation of Toll-Like Receptors 4 (TLR-4) on the plasma membrane, subsequently activating the inflammatory proteins c-Jun N-terminal kinase (JNK), IκB kinase and NF-κB. TNF-α, an adipokine secreted by the adipose tissue, is also capable of activating these inflammatory proteins (Stanley et al., 2011; Stagakis et al., 2012). The inflammatory response induced by these molecules implies the inhibition of eNOS by the reduced expression and kinase activity of the insulin receptor, leading to altered phosphorylation on tyrosine substrates (IRS-1), with a subsequent decrease of the PI3K pathway. This results in reduced NO production by EC; in addition, inhibition of glucose transporter type 4 (GLUT4) translocation to the plasma membrane causes a reduction of glucose uptake in peripheral tissues, thus promoting endothelial dysfunction and peripheral insulin resistance (de Alvaro et al., 2004; Fernández-Veledo et al., 2009).

Furthermore, in response to endothelial injury and inflammation, oxidized lipids from LDL particles (Ox-LDL) accumulate in the endothelial wall of arteries (Dokken, 2008). Monocytes then infiltrate the arterial wall and differentiate into macrophages, which accumulate oxidized lipids to form foam cells. Once formed, foam cells stimulate macrophages and the attraction of T-lymphocytes which, in turn, induce smooth muscle proliferation in the arterial walls and collagen accumulation (Fowler, 2008). Overall, the cellular damage caused by oxidative stress is a trigger for inflammatory stress, which reciprocally stimulates the production of free radicals, closing the pathological cycle underlying progression of diabetes and of its serious vascular complications (Lamb and Goldstein, 2008; Figure 1).

## Mitochondria-Induced Endothelial Cell Dysfunction in Diabetes Progression

**FIGURE 2:**
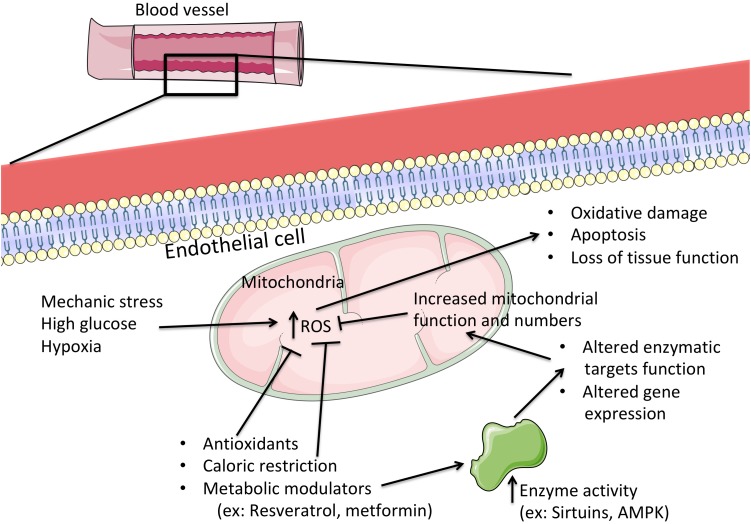
The central role of mitochondria in the progression or prevention of hyperglycemic injury in endothelial cells. When challenged with abnormally high levels of glucose, endothelial cells progressively lose biochemical and functional capabilities, which are perfectly aligned with the loss of mitochondrial function. Here, the excess nutrients lead to a deceleration of metabolic reactions and electron transport in the respiratory chain, leading to a higher generation of reactive oxygen species (ROS). These in turn, when excessive, wreak havoc on cellular structures and biochemical processes, which might ultimately lead to cell death and loss of tissue function. By directly targeting mitochondrial function (for example, by consumption of metabolically active agents or by restriction to caloric intake) one can lead to improved mitochondrial activity by both post-transcriptional and gene expression alterations to mitochondrial function, with concomitant prevention of excessive oxidative stress and preservation of cellular integrity and function. ROS, reactive oxygen species; AMPK, AMP-activated protein kinase.

Endothelial cell mitochondria are highly dynamic organelles in location, number and activity. Typically, angiogenesis leads to a paradoxical increase in both glycolysis and fatty acid oxidation, since glucose is more readily available than oxygen but if fatty acid oxidation is impaired, so is vessel growth (De Bock et al., 2013; Schoors et al., 2015). However, there are no doubts about the effects of hyperglycemia as present in diabetes. Endothelial cells are extremely sensitive to high glucose levels, which leads to apoptosis and loss of tissue function (Triggle et al., 2012) and is associated with elevated mitochondrial fragmentation, altered mitochondrial ultrastructure and increased ROS generation (Pangare and Makino, 2012; Mishiro et al., 2014; Figure 2). Similar results were found in isolated mitochondria from diabetic mouse coronary EC. In fact, accompanying increased mitochondrial fission was an elevated quantity of mitochondrial fission-regulating proteins, such as DRP1 (Makino et al., 2010) and FIS1, the latter in diabetic humans (Shenouda et al., 2011), although this does not appear to be an ubiquitous process (Zhong and Kowluru, 2011). Interestingly, the expression of the mitochondrial biogenic program regulator PGC-1α (peroxisome proliferator-activated receptor gamma coactivator 1-alpha) affects the response of EC mitochondria opposite fashions, depending on the source (Sawada et al., 2014; Craige et al., 2016). We speculate that this might be related to altered mitophagic processes, where the removal of damaged mitochondria is not viable to the cell. Clarification of mitochondrial dynamics and biogenesis in EC diabetic stress will certainly contribute to novel therapeutic approaches. Giving further relevance to the role of mitochondrial function in EC are the numerous studies highlighting how hyperglycemia leads to calcium overload and elevated pro-apoptotic protein BAX content, which result in higher levels of mitochondrial permeability transition induction, ending in apoptosis (Kowluru and Abbas, 2003; Detaille et al., 2005).

Diabetes is typically associated with obesity, where a majority of patients present high levels of LDL-cholesterol which, in its oxidized form, has been shown to contribute to mitochondrial dysfunction and EC apoptotic death (Vindis et al., 2005). Abnormally high generation of mitochondrial ROS in response to increased nutrient availability (as occurs in obesity and diabetes) also has a primordial source, the mitochondrial respiratory chain and, in particular, complexes I and III of the aforementioned chain. Excess nutrients are readily metabolized and generate reducing equivalents for membrane potential generation in the mitochondrial inner membrane (which is primarily used for driving ATP synthesis at Complex V or ATP synthase) (Rolo and Palmeira, 2006). However, the excessive reducing power far outreaches the cell’s ATP needs, as membrane potential (ΔΨ) builds up (Teodoro et al., 2013). As a result, the redox reactions of the mitochondrial respiratory chain decelerate immensely, which results in more reduced respiratory complexes that have no problem in being oxidized by molecular oxygen, leading to heightened ROS generation. As such, a controlled leak of protons back to the mitochondrial matrix presents itself as an attractive target for dealing with excess nutrients. In fact, nature has already developed such a system in the form of uncoupling proteins (UCP). While UCP1 is typically associated (and present) in brown adipose tissue, where it is responsible for this tissue’s famous role in thermogenesis (by dissipation of ΔΨ), other forms of U exist in many other tissues (UCP2 and 3 have been shown to naturally occur in EC), where their role appears to hinge on ROS management and prevention, as one of their more efficient activators are free fatty acids products from triglyceride breakdown (Davis et al., 2008). It has already been demonstrated that UCP2 overexpression in EC reduces fatty acid-caused ROS generation, inflammation and apoptosis (Lee et al., 2005; Koziel et al., 2012), which directly correlates with vasoconstriction, atherosclerotic plaque deposition and ischemic stroke (Szewczyk et al., 2015). Further strengthening these observations is the report that increased ROS generation leads to higher rates of UCP2 expression in EC, which is dependent on the activation of AMP-activated protein kinase (AMPK) and PGC-1α (Valle et al., 2005; Szewczyk et al., 2015). Also, by removing UCP2, ROS, and ΔΨ are increased, which leads to an inflammatory activation and increased EC death (Koziel et al., 2015; Szewczyk et al., 2015). The mechanism by which UCP2 further alters EC response to stress appears to hinge on p53 and its regulation of mitochondria, which is activated by ROS when UCP2 is absent or inactive (Shimasaki et al., 2013). These experimental data seem to gain strength from human diabetic patients carrying a mutation that leads to elevated UCP2 expression, which protects against the increased coronary risk (Cheurfa et al., 2008). Further studies on the matter of UCP2 and EC in diabetes have been reviewed before (Szewczyk et al., 2015).

## Therapeutic Strategies

As briefly reviewed, oxidative stress, inflammation and mitochondrial dysfunction are closely linked with endothelial dysfunction, which is critical in the progression of micro and macrovascular diabetic complications (Figure 1). Thus, therapeutic strategies targeting these biological mechanisms could be pivotal to manage diabetes and its serious complications. Herein, we review the main antioxidant and anti-inflammatory effects associated with some of the most efficient antidiabetic non-pharmacological and pharmacological interventions. A thorough revision of this matter is found below, and a table highlighting the main features of this section is also provided (Table 1).

**Table 1:** Therapeutic strategies targeting some of the biological mechanisms (e.g., oxidative stress, inflammation and mitochondrial dysfunction) underlying endothelial dysfunction, which is critical in the progression of micro and macrovascular diabetic complications.

Therapeutic strategies	Main outcomes	References
**Lifestyle interventions**		
Physical exercise (training)	Improves glycemic control, insulin sensitivity, blood pressure, lipid profile, vascular function (NO availability); antioxidant and anti-inflammatory activity.	Green et al., 2004; Bassuk and Manson, 2005; de Lemos et al., 2007, 2009; Balducci et al., 2010; Kawanishi et al., 2010; Kwon et al., 2011; Teixeira-Lemos et al., 2011; Radak et al., 2013; Robinson et al., 2015.
Dietary interventions	Improves glycemic control, lipid profile and endothelial function; protects against atherosclerosis and reduces cardiovascular risk; antioxidant, anti-inflammatory and antifibrotic properties.	Gey, 1998; Barclay et al., 2007; Aguirre and May, 2008; Abbatecola et al., 2009; Nazıroğlu et al., 2010; Cho et al., 2013; Wang et al., 2013; Yan et al., 2013; Weickert and Pfeiffer, 2018.
**Oral antidiabetics drugs**		
Metformin	Hypoglycemic activity, improvement of insulin sensitivity and cardiovascular risk profile; amelioration of vascular dysfunction (e.g., by restoring NO availability and inhibiting AGEs formation); antioxidant and anti-inflammatory properties.	Ersoy et al., 2008; Yoshida et al., 2009; Sena et al., 2011; Kelly et al., 2012; Krysiak and Okopien, 2012, 2013; Batchuluun et al., 2014; Cheang et al., 2014; Vasamsetti et al., 2015; Triggle and Ding, 2017.
Thiazolidinediones	Hypoglycemic activity, improvement of insulin sensitivity and beneficial modulation of inflammatory, oxidative and endothelial vascular functions.	Cheng and Fantus, 2005; Sourij et al., 2006; Derosa and Sibilla, 2007; Dandona et al., 2008; Orasanu et al., 2008; Zhao et al., 2010; Hamblin et al., 2011; Jin et al., 2016.
Incretin-based therapies	Insulinotropic effects, gastric emptying delaying and reduction of endogenous glucose production by inhibiting glucagon secretion; reduction in blood pressure; improvement in endothelial dysfunction; extra-pancreatic cytoprotective properties, including anti-inflammatory, antioxidant and anti-apoptotic (e.g., against diabetic nephropathy and retinopathy.	Courrèges et al., 2008; Bergenstal et al., 2010; Ferreira et al., 2010; Hattori et al., 2010; Mega et al., 2011, 2014, 2017; Gonçalves et al., 2012, 2014; Lee et al., 2012; Shiraki et al., 2012; Dai et al., 2013; Ishikawa et al., 2014; Marques et al., 2014; Nakamura et al., 2014; Sun et al., 2015.
SGLT-2 inhibitors	Decrease blood glucose levels, body weight and blood pressure; cardiovascular and renal protection via anti-inflammatory and antioxidant effects; attenuate atherosclerotic lesion formation, with reduction of cardiovascular morbidity and mortality.	Hasan et al., 2014; Chilton et al., 2015; Ojima et al., 2015; Scheen, 2015; Tikkanen et al., 2015; Zinman et al., 2015; Leng et al., 2016.
**Mitochondrial-targeting strategies**		
Antioxidants/ ROS scavengers (e.g., MitoQ-TPP and TEMPOL)	Improve vascular prognosis in diabetics by contributing to reduce oxidative stress and blood pressure, thus improving vascular relaxation. Protect against hypertension by preserving EC function, which correlates with improved cardiac function; in addition, prevent inflammation at atherosclerotic plaque sites.	Graham et al., 2009; Dikalova et al., 2010; Mercer et al., 2012.
Caloric restriction	Increases mitochondrial biogenesis and efficiency, and reduces vascular inflammation thus improving EC dysfunction; ameliorates atherosclerosis, diminishes ROS generation, and overall reduces plaque deposition, hypertension and other cardiovascular complications in humans.	Guo et al., 2002; Fontana et al., 2004; Nisoli et al., 2004; López-Lluch et al., 2008; Lefevre et al., 2009; Finckenberg et al., 2012.
Metabolic modulators: (i) Sirtuin 1 activators (e.g., resveratrol) (ii) AMPK activators (e.g., metformin) (iii) PPARγ activators (e.g., pioglitazone)	(i) Decreases p66Shc overexpression induced by hyperglycemia; p66Shc promotes oxidation of several targets in the EC mitochondria, thus contributing to endothelial dysfunction. Resveratrol leads to AMPK activation, eNOS increment, reduction of ROS generation and plaque deposition, culminating in improved EC function. (ii) Inhibit the induction of the mitochondrial permeability transition leading to the prevention of EC apoptosis and endothelial loss of function. (iii) Activates PGC-1α, leading to improved mitochondrial biogenesis in EC, thus contributing to improve endothelial dysfunction.	Wang et al., 2005; Schulz et al., 2008; Csiszar et al., 2009; Fujisawa et al., 2009; Zhou et al., 2011; Price et al., 2012.


### Lifestyle Interventions

Non-pharmacological antidiabetic strategies encompass nutritional guidance and implementation of balanced and low-calorie diets as well as promotion of physical activity. These cornerstone lifestyle interventions could be pivotal not only to prevent diabetes’ appearance and/or progression but also to ameliorate vascular complications, which coincide with a multiplicity of beneficial effects, including antioxidant and anti-inflammatory properties.

#### Physical Exercise (Training)

It has been evidenced that regular physical exercise (training) improves metabolic health due to improvement in glycemic control and insulin sensitivity, as well as due to a positive impact on abdominal circumference and visceral fat (de Lemos et al., 2007, 2009). Regular physical activity also favorably modulates other cardiovascular risk factors associated with T2DM, including blood pressure and lipid profile, demonstrated by the reduction of triglycerides, total-cholesterol and LDL-cholesterol levels together with the increase of high density lipoprotein (HDL)-cholesterol (Bassuk and Manson, 2005; de Lemos et al., 2007). In addition, physical exercise improves vascular function, namely by enhancing NO bioavailability (Green et al., 2004; Kwon et al., 2011). During physical exercise, an increased formation of free radicals is observed, mainly due to increased O_2_ consumption by active tissues (Teixeira-Lemos et al., 2011; Vierck et al., 2012; Radak et al., 2013). Most of this O_2_ consumed is used in the mitochondria for oxidative phosphorylation, where it is reduced to water; however, a small fraction (of about 2–5%) is converted into ROS (Teixeira-Lemos et al., 2011). Chronic physical activity of moderate intensity (training) positively alters the oxidative homeostasis of cells and tissues due to a reduction of basal levels of oxidative damage and to an increase in oxidative stress resistance (Cooper et al., 2002). In fact, regular exercise leads to an adaptation in antioxidant capacity, as viewed by the increment of superoxide dismutase and glutathione peroxidase activities, thus protecting cells against damage caused by oxidative stress (Teixeira-Lemos et al., 2011; Radak et al., 2013).

Furthermore, long-term training is an efficient anti-inflammatory measure, expressed by the reduction of pro-inflammatory mediators, such as CRP, interleukin-6 and TNF-α, and simultaneously by the increment of anti-inflammatory cytokines such as IL-4, IL-10 and adiponectin (Balducci et al., 2010; Teixeira-Lemos et al., 2011; Radak et al., 2013). One hypothesized mechanism through which training might exert an anti-inflammatory activity may be related to the modulation of TLR-dependent pathways (Kawanishi et al., 2010; Robinson et al., 2015) as viewed by the reduced expression of TLR2 and TLR4 in monocytes, lymphocyte and neutrophils in obese adults with a higher risk for developing T2DM after training of moderate intensity (Robinson et al., 2015).

#### Dietary Interventions

Lifestyle intervention programs in T2DM patients, especially in those who are obese, have been mainly focused on the reduction of caloric intake and on body weight loss, which seem *per se* to have a major beneficial impact on glycemic control and cardiovascular risk (Buse et al., 2006). Several studies have been carried out to identify the perfect combination of macronutrients able to prevent the onset of CVD; nevertheless, the best combination of proteins, carbohydrates and lipids varies according to the individual, which makes it difficult to define a universal food plan for this type of patients (Franz et al., 2004; Grundy et al., 2005). However, it is known that some foods (and nutrients contained therein) exert anti-diabetic and vasoprotective properties, in particular because they have antioxidant and anti-inflammatory effects.

Numerous studies have shown that diets rich in whole grains, omega (ω3) fatty acids and fibers, associated with a low consumption of *trans* fatty acids carbohydrates and cholesterol, are recommended strategies to improve the lipid profile and to reduce cardiovascular risk in T2DM patients with a high glycemic index (Barclay et al., 2007; Abbatecola et al., 2009; Cho et al., 2013; Weickert and Pfeiffer, 2018). More precisely, there is a wide variety of antioxidative substances found in food, mainly in fruits and vegetables, which can synergistically act in the protection of cells and tissues (Blomhoff, 2005; Halvorsen et al., 2006). Several epidemiological studies have suggested a direct association between vitamin E intake and reduction of cardiovascular morbidity and mortality (Wang et al., 2013), although limited information is available regarding the impact of vitamin E supplementation on T2DM patients (Boshtam et al., 2005; Giannini et al., 2007). On the other hand, a combination of vitamins C and E seems to be an effective strategy due to inhibition of lipid peroxidation and protection against DNA damage, as previously reported (Gey, 1998; Nazıroğlu et al., 2010). Vitamin C can afford protection in several types of vascular cells involved in the process of atherosclerosis: ascorbate helps to prevent endothelial dysfunction, stimulates the synthesis of type IV collagen and increases proliferation, while also inhibiting differentiation and proliferation of vascular smooth muscle cells in areas of injury and reducing oxidative stress in macrophages (Aguirre and May, 2008). Antioxidants can inhibit lipid peroxidation directly by scavenging the peroxide radicals and indirectly by regenerating the active form of other antioxidant compounds, like vitamin E, flavonoids and glutathione (Aguirre and May, 2008).

Consumption of ω3-polyunsaturated fatty acids (PUFAs) seems to provide cardioprotection in diabetic conditions due to pleiotropic properties, including those of an antioxidant, anti-inflammatory and antifibrotic nature. Regarding the impact on inflammation, ω3-PUFAs were associated with attenuation of both TLR4 and TNF-α-mediated pro-inflammatory signaling in macrophages and inhibition of the inflammasome via effects on NLRP3 in high-fat diet (HFD)-induced diabetic mice (Yan et al., 2013).

### Oral Antidiabetics Drugs

#### Metformin

Metformin is a first-line pharmacological treatment for most T2DM patients. This anti-hyperglycemic agent is an activator of AMPK and suppresses hepatic glucose synthesis and improves insulin sensitivity by enhancing insulin-stimulated peripheral glucose uptake (Yoshida et al., 2009). In addition to its hypoglycemic effect, other beneficial effects of this drug are being studied, including its role on the prevention of vascular complications. Clinical trials that have enrolled overweight adult patients with or without T2DM support the decrease of cardiovascular risk profile induced by metformin (De Jager et al., 2005; Ersoy et al., 2008; Kelly et al., 2012); however, the underlying mechanism of metformin’s cardioprotective actions remains to be fully understood. Emerging evidence suggests that metformin boasts both direct and indirect antioxidant and anti-inflammatory properties (Sena et al., 2011; Krysiak and Okopien, 2012, 2013; Vasamsetti et al., 2015). The activation of AMPK pathways plays a pivotal role on its pharmacological effects and might explain the variety of pleiotropic actions of this drug. In fact, several mechanisms that explain metformin’s beneficial actions have been proposed, including NF-kB inhibition, NO production incrementation and inhibition of AGEs formation.

The antioxidative effect of metformin may be related with the reduction of DAG levels, inhibition of PKC translocation to the cellular membrane, and suppression of the NADPH oxidase activity, leading to reduced ROS production (Piwkowska et al., 2010; Batchuluun et al., 2014). In obese mice fed with a HFD, treatment with metformin improved endothelial function by reducing endoplasmic reticulum stress, superoxide production and by increasing NO bioavailability (Cheang et al., 2014). Metformin has been shown to directly inhibit ROS production from complex I (NADH: ubiquinone oxidoreductase) of the mitochondrial electron transport chain (Owen et al., 2000) and to increase the AMP/ATP ratio. Moreover, Sartoretto et al. (2005) reported that metformin increases NO activity, but not expression, and that it improves microvascular reactivity to histamine, bradykinin or acetylcholine of arterioles and venules in T2DM animal models. A number of basic and clinical studies investigated the effect of metformin on endothelium-dependent vascular function (Mather et al., 2001; Sena et al., 2011; Triggle and Ding, 2017). In particular, Sena et al. (2011) have reported that metformin restored endothelial function by enhancing NO bioavailability and reducing oxidative stress and inflammation in the aortic rings of normal and high fat-fed diabetic GK rats. The authors showed that metformin treatment reduced monocyte chemoattractant protein-1 (MCP-1/CCL2) levels that were increased in T2DM rats, indicating an inhibition of early inflammation in the aorta (Sena et al., 2011).

Previous studies have described that metformin inhibits proinflammatory responses in vascular endothelial and VSMCs (Hattori et al., 2006; Isoda et al., 2006). Hattori et al. (2006) have reported that metformin inhibited cytokine-induced NF-kB activation via AMPK activation in human vascular EC. Another report demonstrated that metformin may attenuate Ox-LDL-induced proinflammatory responses in monocytes and macrophages and inhibit monocyte-to-macrophage differentiation (Huangfu et al., 2018). Vasamsetti et al. (2015) reported that metformin inhibits monocyte-to-macrophage differentiation by decreasing STAT3 phosphorylation through increased AMPK activation and leads to a reduction of proinflammatory cytokine production. Furthermore, clinical studies also suggest that metformin may modulate the inflammatory status as viewed by the reduction of several proinflammatory cytokines (Krysiak and Okopien, 2012, 2013). Krysiak and Okopien (2013) demonstrated that metformin could reduce the monocyte secretion of TNF-α, IL-1β, IL-6, MCP-1, and IL-8, as well as plasma CRP levels, in patients with impaired fasting glucose. Another study with obese diabetic patients showed that after 12 weeks of metformin therapy, there was a decrease in PAI-1 and in vascular endothelial growth factor (VEGF) (Ersoy et al., 2008). Moreover, De Jager et al. (2005) also observed, in patients with T2DM, that treatment with metformin for 16 weeks reduced levels of plasma VCAM-1, soluble E-selectin, PAI-1, and von Willebrand factor (vWF), whereas markers of inflammation were unaffected. Thus, in clinical trials the anti-inflammatory effects of this drug remain to be fully clarified.

#### Thiazolidinediones

Thiazolidinediones (TZDs), including rosiglitazone and pioglitazone, are a class of drugs known to improve insulin sensitivity in peripheral tissues. These drugs bind to and activate the peroxisome proliferator-activated receptor gamma (PPARγ), which is found in the liver, muscle, heart, kidney, and adipose tissue and is involved in the regulation of expression of insulin-sensitive genes, which are crucial to glucose and lipid metabolism. Apart from the hypoglycemic effects, TZDs have showed an ability to modulate inflammatory, oxidative and vascular functions.

The anti-inflammatory action of TZDs has been suggested by the increment of adiponectin and parallel reduction of cytokine production from the adipose tissue, such as TNF-α and resistin (Cheng and Fantus, 2005). Furthermore, it was suggested that pioglitazone decreases inflammation partly through inhibiting AGE-induced classical macrophage polarization in diabetic HFD fed mice (Jin et al., 2016). A meta-analysis showed that pioglitazone and rosiglitazone significantly decreased serum CRP levels in subjects with and without diabetes, irrespective of the effects on glycemia (Zhao et al., 2010). Previous studies have suggested an improved endothelial function with TZD treatment (Derosa and Sibilla, 2007; Dandona et al., 2008). In *in vitro* and *in vivo* studies, pioglitazone protects against oxidative stress, reduces blood pressure and decreases VCAM-1 expression on EC through modulation of NF-κB activity via a PPARα-dependent mechanism (Orasanu et al., 2008; Hamblin et al., 2011). Concurrent with this, pioglitazone was also shown to improve endothelial function in non-diabetic individuals with coronary artery disease, suggesting that pioglitazone exerts a direct effect on the endothelium (Sourij et al., 2006). In addition, in obese and diabetic patients, pioglitazone has been shown to reduce arterial stiffness and reduce blood pressure and CRP levels, independently of changes in glycemic control (Satoh et al., 2003; Clarke et al., 2017). Although there are several studies describing the beneficial vascular effects of PPAR agonists, including the PROACTIVE (Prospective Pioglitazone Clinical Trial in Macrovascular Events) trial, the cardiovascular effects of TZDs are still not well understood, and current clinical evidence leaves this hypothesis unproven. The recognition of an increased risk of myocardial infarction and heart failure in some patients using rosiglitazone therapy (Nissen and Wolski, 2007), which caused its withdrawal in Europe and limited use in the United States, added controversy to this subject, leading to a need for further clarification.

#### Incretin-Based Therapies

Glucagon-like peptide-1 (GLP-1) is an incretin hormone primarily synthesized by the endocrine L cells of the gastrointestinal tract during and after food intake and stimulates glucose-dependent insulin secretion by pancreatic β cells. The insulin secretory response of incretins is known as the incretin effect, and accounts for at least 50% of postprandial insulin secretion. In addition to its insulinotropic effects, GLP-1 delays gastric emptying and also reduces endogenous glucose production by inhibiting glucagon secretion by pancreatic α-cells (Triplitt et al., 2006). This incretin is rapidly metabolized by the ubiquitous enzyme dipeptidyl peptidase-IV (DPP-IV) to inactive metabolites, which are then eliminated by the urine (Ranganath, 2008), resulting in the inactivation of the incretin effect (Holst, 2007). The recognition, and further characterization of an impaired incretin effect (known as the incretin defect) in diabetic patients was the base for the development of a new class of antidiabetic agents, the incretin-based therapies, which includes DPP-IV inhibitors and GLP-1 receptor (GLP-1R) agonists (Gallwitz, 2005). Over the last years, extra-pancreatic protective effects, behind glucose-insulin control, have been suggested in distinct vascular conditions (Scirica et al., 2013; Godinho et al., 2015).

In addition to regulating glucose and metabolic control, GLP-1 has a potential beneficial effect on multiple pathways involved in atherogenesis. Although the mechanisms of vascular effect are still unclear, it seems that the protective action of GLP-1 may be related to an improvement in endothelial dysfunction through its anti-inflammatory and antioxidant effects (Nyström et al., 2004). Several studies performed in T2DM patients showed a positive impact of GLP-1R agonist treatment in endothelial function (Nikolaidis et al., 2004; Sokos et al., 2006). Liraglutide and exenatide therapy were able to reduce the levels of PAI-1 inhibitor, a regulator of plasminogen activation implicated in EC dysfunction (Courrèges et al., 2008; Liu et al., 2009; Bergenstal et al., 2010). Although the exact mechanisms remain to be fully elucidated, several studies reporting on treatment with these two GLP-1R agonists have consistently demonstrated a reduction in blood pressure in patients with T2DM as well as a reduction in brain natriuretic peptide (BNP) levels (Courrèges et al., 2008; Bergenstal et al., 2010; Sun et al., 2015). Moreover, in a human vascular endothelial cell line, liraglutide inhibited TNF-α, fibrinolysis inhibitor PAI-1, and the mRNA and protein levels of VCAM-1 and ICAM-1 (Liu et al., 2009). Likewise, it has been reported that liraglutide exerts marked antioxidant and anti-inflammatory effects on vascular EC by increasing NO production, with inhibition of PKC-α, NADPH oxidase, NF-κB and JNK signaling, while also leading to the overexpression of superoxide dismutase (SOD) and catalase protective antioxidant enzymes (Hattori et al., 2010; Shiraki et al., 2012). Additionally, also in *in vitro* studies, lipopolysaccharide (LPS)-stimulated inflammatory responses were inhibited by exendin-4, a GLP-1 analog, in cardiomyoblasts (Chen et al., 2012) and in human peripheral mononuclear cells (PBMCs) (Hogan et al., 2014). In ob/ob mice, treatment with recombinant adenovirus producing GLP-1 inhibited macrophage infiltration and adipose tissue expression and production of IL-6, TNF-α, and MCP-1 (Lee et al., 2012). Dai et al. (2013) reported that liraglutide protects against atherogenesis by the reduction of Ox-LDL-induced mitochondrial ROS in human aortic VSMCs. In addition, these inhibitory effects were abrogated by the overexpression of lectin-like oxidized low-density lipoprotein scavenger receptor-1 (LOX-1), suggesting that LOX-1 plays an important role in the antioxidant effects of GLP-1R agonists.

Dipeptidyl peptidase-IV inhibitors, another group of incretin-based therapies, increase endogenous incretin levels availability (such as GLP-1) and exert several insulinotropic effects that contribute to maintain normal glucose levels (Godinho et al., 2015). In particular, DPP-IV inhibitors are able to enhance glucose-stimulated insulin secretion, inhibit glucagon secretion and promote beta-cell proliferation and survival (Brubaker and Drucker, 2004). Apart from the insulin-dependent effects, DPP-IV inhibitors, namely sitagliptin (the first drug of this new class of antidiabetic agents), exert extra-pancreatic cytoprotective properties. Our group has already demonstrated antioxidant and anti-inflammatory effects of sitagliptin in animal models of diabetic nephropathy and diabetic retinopathy (Ferreira et al., 2010; Mega et al., 2011, 2014, 2017; Gonçalves et al., 2012, 2014; Marques et al., 2014). In models of type 1 and type 2 diabetes, sitagliptin was able to ameliorate diabetic retinopathy by preventing nitrosative stress, inflammation and apoptosis in retinal cells and by exerting beneficial effects on the blood retinal barrier (Gonçalves et al., 2012). In the Zucker Diabetic Fatty (ZDF) rat, a model of obese T2DM, sitagliptin ameliorated diabetic nephropathy, which was accompanied by an antioxidant effect and by a significant reduction in inflammatory state and cell death by apoptosis (Mega et al., 2011, 2017; Marques et al., 2014).

Other studies in both animals and humans have suggested similar protective properties in other tissues and conditions, including a positive impact on vascular endothelium with anti-atherosclerotic action (Ishikawa et al., 2014; Nakamura et al., 2014), usually based on the anti-inflammatory activity of these antidiabetic agents (Lee et al., 2012; Satoh-Asahara et al., 2013). In fact, T2DM patients treated during 12 weeks with sitagliptin presented a reduction in circulating inflammatory markers, like CRP and IL-6 and reduced monocyte expression of mRNA transcripts associated with inflammation (Makdissi et al., 2012). Furthermore, treatment with sitagliptin improved the inflammatory state, vascular endothelial function and prevented the progression of carotid atherosclerosis in a dose-dependent manner independent of its glucose-lowering effects (Ayaori et al., 2013). Considering the direct effects regarding the glycemic control and the pleiotropic effects on extra-pancreatic tissues, DPP-IV inhibitors as well as GLP-1R agonists are promising options for managing diabetes and vascular complications.

#### SGLT-2 Inhibitors

Selective sodium/glucose co-transporter 2 (SGLT-2), is a high-capacity, low-affinity glucose transport protein, which is primarily found in the kidney but also in the intestine (Hasan et al., 2014). This glucose transporter is responsible for about 90% of glucose reabsorption in the kidney (Vallon, 2011). Empagliflozin, canagliflozin, and dapagliflozin are SGLT-2 inhibitors, also known as gliflozins, currently available in Europe and in the United States for diabetes management. In diabetic conditions, the kidneys increase expression of SGLT-2 and, unlike the liver, increased glucose ingestion elevates kidney gluconeogenesis even in diabetic patients (Meyer et al., 1998; Hasan et al., 2014). SGLT-2 inhibitors exert their effect on the kidneys, preventing reabsorption of glucose from the proximal tubules via SGLT-2 (Scheen, 2015). Clinical trials of SGLT-2 inhibitors have shown that these drugs decreased glucose levels independent of insulin (Scheen, 2015). SGLT-2 inhibitors may also have additional benefits related with weight loss and with reductions in blood pressure that occur because of the osmotic effect of glucose excretion and subsequent inhibition of the renin-angiotensin system (Scheen, 2015). Recently, a few studies indicated that SGLT-2 inhibitors may exert their cardiovascular and renal protection via anti-inflammatory and antioxidative effects (Ojima et al., 2015). Leng et al. (2016) found that dapagliflozin can attenuate the formation of atherosclerotic lesions, increase the stability of lesions, reduce the production of IL-1β by macrophage infiltration, and decrease mitochondrial ROS generation. These effects may be associated with an inhibitory effect on the NLRP3 inflammasome in diabetic atherosclerosis, which provides further evidence for its benefits in diabetic patients. Moreover, also in diabetic rats, empagliflozin was shown to improve hyperglycemia, reduce urinary excretion levels of tubular injury markers, decrease expression levels of oxidative stress biomarkers (AGEs and RAGE), and reduce inflammatory and fibrotic markers in the kidney, including MCP-1, ICAM-1, PAI-1, TGF-β (Ojima et al., 2015). The authors suggest that a blockade of SGLT-2 by empagliflozin might protect proximal tubular cells from glucotoxicity in diabetic nephropathy partly via suppression of the AGE-RAGE-mediated oxidative stress generation. Furthermore, empagliflozin given to patients with T2DM was shown to reduce both blood pressure and arterial stiffness (Chilton et al., 2015; Tikkanen et al., 2015), and data from the EMPA-REG OUTCOME (Empagliflozin Cardiovascular Outcome Event Trial in Type 2 Diabetes Mellitus Patients–Removing Excess Glucose) trial showed that empagliflozin is the first antidiabetic drug compound to conclusively reduce cardiovascular morbidity and mortality (Zinman et al., 2015).

### Mitochondrial-Targeting Strategies

By targeting mitochondrial ROS generation, it is possible to increase endothelial activity and improve endothelial dysfunction in diabetes (Figure 2). For example, it has been shown that the nicotinamide adenine dinucleotide phosphate oxidase 4 (NOX4) enzyme regulates mitochondrial ROS generation, leading to vessel relaxation and lower blood pressure (Santillo et al., 2015). This is further confirmed by the fact that higher levels of low-density lipoproteins, typically associated with obesity and diabetes, increase NOX activity, which leads to increased oxidative stress and cell death (Li et al., 2016). Also, NOX activity has been implicated in altered angiogenesis and increased susceptibility to hypoxia and stroke (Amanso and Griendling, 2012; Caja and Enríquez, 2017). Interestingly, NOX4 exists in EC and, in certain conditions, is present within mitochondria, where it can block the respiratory chain complex I, leading to increased ROS generation (Kozieł et al., 2013) and elevating the interest in this enzyme for diabetic-associated ROS management (Touyz and Montezano, 2012). Another regulator of mitochondrial ROS, with clear implications for endothelial dysfunction, is p66Shc, which responds to high glucose by migrating to mitochondria and inducing oxidation of key effectors, leading to cell death and loss of tissue function (Camici et al., 2007; Paneni et al., 2012). In particular, p66Shc can oxidize reduced cytochrome *c*, which accumulates in anoxic conditions, a prevalent situation in diabetes in certain tissues where cell enlargement (ex: adipocytes) is present (Giorgio et al., 2005). The NAD^+^-dependent deacetylase Sirtuin 1 is a known regulator of mitochondrial function and cellular homeostasis (Price et al., 2012). It has already been demonstrated that EC-specific Sirtuin 1 activation decreases p66Shc overexpression by hyperglycemia, which is predictably related to improved endothelial function (Zhou et al., 2011).

Another factor of ROS-mediated EC injury is the oxidation of NO by ROS. Not only is NO removed (leading to increased vasoconstriction and thus hypoxia and further ROS generation, as well as mechanic injury to the vessel due to elevated blood pressure) but NO synthase activity is also compromised, resulting in even further ROS generation (Forstermann and Münzel, 2006). As such, antioxidant therapy or elevation of the natural antioxidant defenses in mitochondria has been shown to significantly improve vascular diabetic prognosis by contributing to reduced oxidative stress, vascular relaxation and reduced blood pressure (Dikalova et al., 2010).

However, since ROS has transitioned from simply toxic agents toward a signaling function, a careful approach to indiscriminate antioxidant therapy must be taken. In fact, mitochondrial ROS are implicated in normal physiological roles of EC, such as shear-stress-induced vasodilation, hypoxia handling, autophagy, and inflammation (Caja and Enríquez, 2017). Nevertheless, pathological surges in ROS generation are undoubtedly connected to loss of vascular function (Brownlee, 2001; Yu et al., 2006). This has been demonstrated by the use of mitochondrial-targeting ROS scavengers. Mitoquinone (mitoQ) attached to triphenylphosphonium (TPP) protected against hypertension by preserving EC function, which correlates with improved cardiac function (Graham et al., 2009), while it can also prevent inflammation at atherosclerotic plaque sites (Mercer et al., 2012). Similarly, the use of mitochondria-targeting TEMPOL, a known ROS scavenger, yielded similar results by decreasing hypertension (Dikalova et al., 2010).

A different approach hinges on preventing heightened ROS generation by limiting nutrient availability, whether by caloric restriction or agents that mime its effects. Unsurprisingly, calorie restriction increases mitochondrial biogenesis and efficiency (Nisoli et al., 2004; López-Lluch et al., 2008), which can be harnessed for management of diabetic-induced EC dysfunction. In fact, this is far from a novel idea, since Young and collaborators have shown that calorie restriction improves blood pressure in hypertense rats (Young et al., 1978), but it was recently confirmed that it reduces cardiac hypertrophy and vascular inflammation, in part by targeting mitochondrial function (Finckenberg et al., 2012). This is further demonstrated by the fact that caloric intake reduction improves atherosclerosis, diminishes ROS generation (Guo et al., 2002), and overall reduces plaque deposition, hypertension and other cardiovascular complications in humans (Fontana et al., 2004; Lefevre et al., 2009). These effects, unsurprisingly, involve the modulation of the activity of many previously discussed agents, such as Sirtuin 1 and AMPK, so much so that their activity modulating compounds are widely considered calorie restriction mimetics (Fontana et al., 2010). For example, the widely consensual Sirtuin 1 activator resveratrol (Price et al., 2012) leads not only to AMPK activation, as the activity of this protein elevates eNOS, but also reduces ROS generation and plaque deposition, culminating in improved EC function (Wang et al., 2005; Csiszar et al., 2009). Similarly, the known AMPK activator metformin inhibits the induction of the mitochondrial permeability transition, leading to the prevention of EC apoptosis and endothelial loss of function (Schulz et al., 2008). Finally, by activating PPARγ, the TZD pioglitazone activates PGC-1α, leading to improved mitochondrial biogenesis in EC. PGC-1α can also be activated by both AMPK and Sirtuin 1, which highlights the interaction and interconnection of these metabolic agents and pathways (Fujisawa et al., 2009).

The most common class of ion-selective channels are potassium transmembrane channels (Szewczyk et al., 2015). While the majority of these channels are present in the plasma membrane, they are also present in the inner mitochondrial membrane, where they regulate potassium fluxes and thus modulate various mitochondrial functions and parameters, such as membrane potential, ATP and ROS generation, mitochondrial volume and calcium import (Bernardi, 1999; Szabo and Zoratti, 2014). Given the accumulation of negative charges in the mitochondrial matrix, the positively charged potassium ions enter the mitochondria in favor of the gradient and cause a dissipation of membrane potential and thus decrease ROS generation. As expected, EC mitochondria have potassium channels where their activation decreases ΔΨ and thus lead to vasodilation (Katakam et al., 2013), an effect that might be described by the apparent physical coupling of potassium channels with the respiratory chain (Bednarczyk et al., 2013).

## Conclusion and Perspectives

Vascular complications represent the major cause of morbidity and mortality in T2DM patients and are responsible for the lower life expectancy of these subjects. Given the complexity of the mechanisms involved in disease appearance and progression, it is unlikely that a single therapeutic measure could efficiently control all the factors underlying the slow but consistent deregulation of micro and macro vascular beds. While some patients may greatly benefit from interventions based on changes in lifestyle habits, for the majority of T2DM subjects, pharmacological interventions are unavoidable and crucial. However, apart from the most recent trial with the SGLT-2 inhibitor empagliflozin (EMPA-REG OUTCOME), the extensive list of oral antidiabetic drugs already available for treating T2DM patients has failed to show consistent reduction in cardiovascular mortality, despite collectively, in mono and/or combined therapy, being able to provide good glycemic control. This evidence suggests that we still need to improve the knowledge about disease and complications in order to be able to develop newer targets of intervention. In order to circumvent the current limitations and improve our capacity to fight T2DM and its serious complications, there is growing interest in looking for some of the mechanisms that play a major role, such as hyperglycemia and hyperlipidemia-evoked oxidative stress, mitochondrial dysfunction and inflammation. By better controlling the causes of vascular disease(s) and targeting the mechanisms involved, with older or newer agents, we can expect improvements in the cardiovascular morbidity and mortality outcomes of T2DM patients.

## Author Contributions

All authors participated in the writing and editing of this review article. All authors read and approved the final version of the manuscript.

## Conflict of Interest Statement

The authors declare that the research was conducted in the absence of any commercial or financial relationships that could be construed as a potential conflict of interest. The handling Editor declared a shared affiliation, though no other collaboration, with the authors at the time of review.
